# Testicular degeneration in a jaguarundi (*Puma yagouaroundi*): a histological and functional case study

**DOI:** 10.1007/s11259-026-11098-3

**Published:** 2026-02-09

**Authors:** César Augusto Pinzón-Osorio, Daniela Cortes-Beltran, Dionet Keny Bellido-Quispe, Rafael Gianella Mondadori, Harvey Lozano-Márquez, Jorge Zambrano-Varón, Claudia Jiménez-Escobar

**Affiliations:** 1 Grupo de Investigación en Reproducción Animal y Salud de Hato, Facultad de Medicina Veterinaria y Zootecnia, Universidad Nacional de Colombia (UNAL), Bogotá, DC Colombia; 2 Laboratório de Fisiopatologia e Biotécnicas da Reprodução Animal (FiBRA), Universidade Federal de Pelotas (UFPel), Pelotas, RS Brazil; 3 Department of Large Animal Medicine and Surgery, College of Veterinary Medicine, University of Georgia, GA 30602 Athens, USA; 4 Laboratório de Genômica Estrutural, Universidade Federal de Pelotas (UFPel), Pelotas, RS Brazil; 5 Instituto de Biologia, Grupo Fisiopatologia e Biotécnicas da Reprodução Animal (FiBRA), Universidade Federal de Pelotas (UFPel), Pelotas, RS Brazil

**Keywords:** Subfertility, Spermatogenesis, Sperm, Testis, Wild felids

## Abstract

A free-ranging adult male jaguarundi (*Puma yagouaroundi*) was referred to a wildlife rehabilitation center after sustaining blunt trauma caused by a vehicle collision and died one month later. Following death due to trauma-related complications, a targeted post-mortem examination focused on the reproductive system and laboratory analyses were conducted to evaluate potential reproductive abnormalities. Macroscopic examination of the testis revealed no external alterations. However, histopathological evaluation confirmed moderate bilateral testicular degeneration, characterized by reduced seminiferous tubule diameter and epithelial height, vacuolization, germ cell loss, and premature shedding of immature cells into the lumen and epididymis. Approximately 70% of tubules were atrophic, although some retained limited spermatogenic activity. Epididymal sperm analysis revealed poor motility, reduced viability, and impaired plasma and acrosomal membrane integrity, along with a high proportion of morphological abnormalities. These findings were consistent with impaired spermatogenesis and functional compromise. No inflammatory infiltrates were observed, supporting a non-inflammatory, likely trauma-related etiology. To our knowledge, this is the first report documenting testicular degeneration in *P. yagouaroundi*. These findings contribute to understanding reproductive pathology in wild felids and underscore the importance of routine reproductive assessment in conservation efforts.

**Supplementary Information:**

The online version contains supplementary material available at 10.1007/s11259-026-11098-3.

## Background

Testicular degeneration (TD) is a reproductive pathology characterized by the deterioration of testicular tissue, potentially affecting spermatogenesis and overall fertility (Turner 2007; Barth 2014). It is a relatively uncommon pathology in felids (Elcock and Schoning 1984; Foster et al. 1996; Sigurðardóttir et al. 2001; Foster 2012; Chaudhary et al. 2024), although research in domestic cats shows a prevalence of TD between 0.15% and 10% (Elcock and Schoning 1984; Axnér et al. 1996; Axnér and Linde Forsberg 2007; Axner 2008). Reports of this condition in wild felids are notably absent from the literature. This knowledge gap hinders our understanding of how such reproductive disorders may influence the long-term viability of wild populations.

The jaguarundi (*Puma yagouaroundi*; É. Geoffroy Saint-Hilaire, 1803) is a rare and understudied small neotropical feline ranging from the southern United States to northern Argentina (Giordano 2016; Filoni et al. 2017). Despite it conservation status varying from “least concern” to “vulnerable” depending on the region (Giordano 2016; Abra et al. 2021; Fischer Meinert et al. 2021), the species faces population decline due to habitat loss and fragmentation (Andrews et al. 2019; Abra et al. 2021). This situation underscores the urgency of conservation efforts. However, reproduction studies on jaguarundis are limited, with only a few reports on their seminal characteristics (Swanson et al. 2007; Madrigal-Valverde et al. 2019; Ceregatti and Feitosa 2023; da Costa Estrela et al. 2023).

The present study aims to document and analyze the first known case of TD in a jaguarundi. We provide a comprehensive assessment of sperm morphology, and functionality from fresh epididymal samples, complemented by histopathological evaluation. This case contributes to the limited understanding of reproductive pathologies in wild felids and highlights the importance of identifying subclinical reproductive disorders that may affect individual fertility and compromise the success of *ex situ* conservation programs.

## Case presentation

An adult male jaguarundi (*Puma yagouaroundi*) was received at the Reproductive Biotechnology Laboratory, Universidad Nacional de Colombia, Savannah of Bogotá (4º11’31.3’’ to 5º16’36.6’’ N, 73º21’33.4’’ to 74º26’28.3’’ W) as part of a post-mortem genetic resource preservation initiative. The 5.5 kg male (estimated age: 3–5 years) had sustained traumatic injuries from a car blunt trauma and was referred by the university’s Wild Animal Rescue and Rehabilitation Unit (URRAS). Despite receiving veterinary care, the animal did not recover and died approximately one month later. According to the clinical history and evolution during hospitalization, death was attributed to presumed complications associated with severe blunt trauma sustained during the vehicle collision.

Following death, post-mortem procedures were directed toward reproductive germplasm recovery, prioritizing the rapid collection of testes and epididymides for sperm retrieval and subsequent analyses. Upon external examination, no gross abnormalities were observed in the scrotal region. Immediately after arrival at the laboratory, the testis, epididymis, and spermatic cords were dissected and processed. Testicular measurements were taken using a graduated caliper, and testicular volume was calculated based on the ellipsoid formula: 4/3π × (length/2) × (width/2) × (height/2) (Leme et al. 2018), yielding a volume of 1667 ± 25.0 mm³.

Sperm was retrieved by retrograde flushing of the epididymal tail and proximal vas deferens using a blunted 25-gauge needle connected to a 3-mL syringe containing 1.5 mL of pre-warmed (37 °C) Tris–fructose–citrate solution (295 mOsm/L, pH 6.6) (Ponglowhapan et al. 2006). The suspension was maintained at 37 °C in sterile tubes. The interval between death and orchiectomy was approximately 15 min, and between orchiectomy and sperm recovery was approximately 25 min.

Sperm concentration, determined using a Bürker hemocytometer (Gutiérrez-Reinoso and García-Herreros 2016), was 21 ± 11.04 × 10⁶ sperm/mL. Sperm morphology was analyzed through eosin-nigrosin staining, with 200 cells per sample examined under oil immersion microscopy (Axnér et al. 1999). Only 31.5 ± 2.12% of sperm were morphologically normal. Notably, proximal and distal cytoplasmic droplets were elevated (15.0 ± 1.41% and 7.5 ± 3.54%, respectively) (Suppl. Table 1, Suppl. Figure 1). Functional sperm parameters including motility, vigor, viability, SPMF, and PAMI, were assessed at 0-, 15-, 30-, and 45-minutes post-collection by sperm resilience and thermal sensitivity, a thermo-resistance test (TRT) (CBRA 2013).

**Fig. 1:**
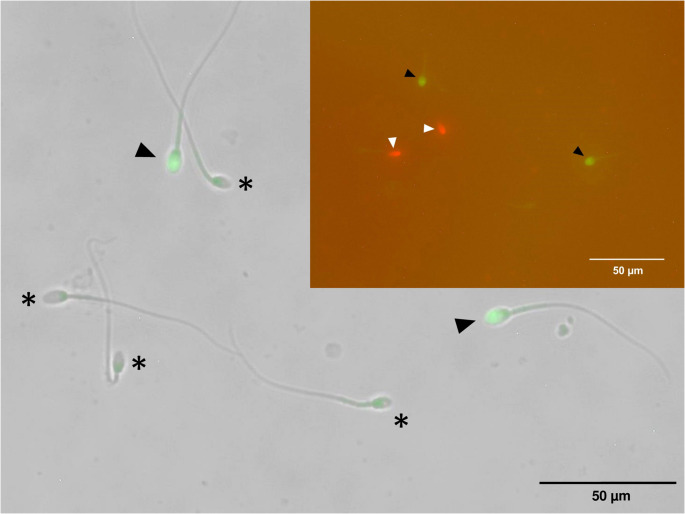
Microphotographs of epididymal sperm from jaguarundi (*Puma yagouaroundi*) to evaluate plasma and acrosomal membrane integrity (PI/PNA dying). Asterisks indicate sperm with intact plasma and acrosomal membranes (PI−/PNA−). Black arrowheads denote sperm with intact plasma membranes but compromised acrosomal membranes (PI−/PNA+). Inset: Black arrowheads show sperm with intact plasma membranes but compromised acrosomal membranes (PI−/PNA+), and white arrowheads show sperm with compromised plasma membranes and intact acrosomal membranes (PI+/PNA−).

At the initial time point (0 min), sperm exhibited progressive motility and vigor of 11.6 ± 1.6 and 0.6 ± 0.3, respectively. These parameters deteriorated rapidly over the 45-minute observation period, with progressive motility dropping below 10% and vigor decreasing to nearly 1 (Suppl. Figure 2). Plasma membrane functionality was assessed using the hypoosmotic swelling (HOS) test (Revell and Mrode 1994), which revealed approximately 35% at initial evaluation (0 min) to just above 10% by 45 min. Viability was determined using eosin-nigrosin staining (Agarwal et al. 2016), showing a decline from 65% to under 20%. Integrity of plasma and acrosomal membranes was assessed via dual fluorescent staining with propidium iodide and PNA-Alexa Fluor 488 (Hernández-Avilés et al. 2018), revealing a reduction in acrosome-intact sperm from approximately 50% at initial evaluation to around 10% by 45 min (Suppl. Figure 2) (Fig. 1).

**Fig. 2:**
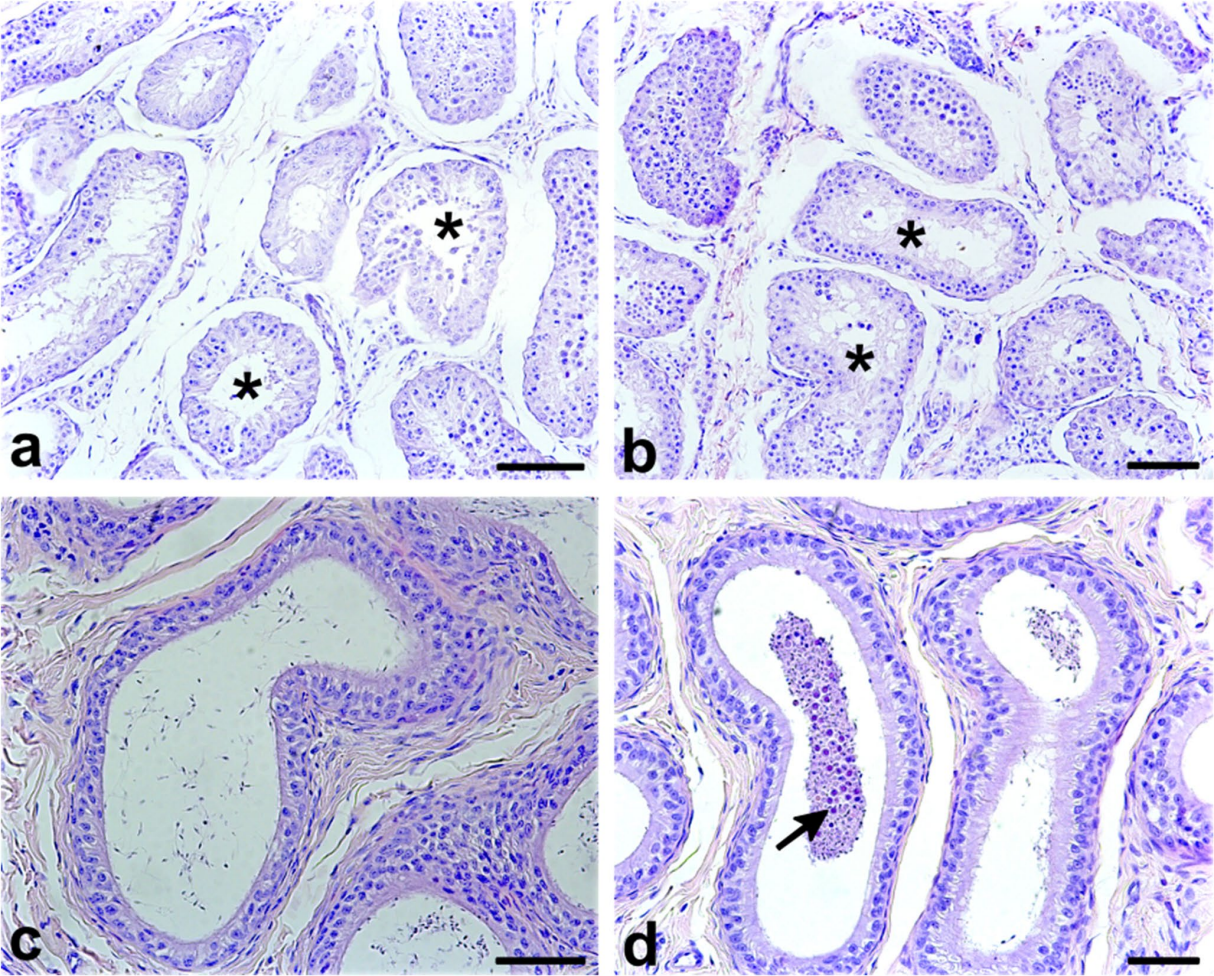
Representative photomicrographs of the seminiferous tubules and epididymis from a male jaguarundi (*Puma yagouaroundi*) diagnosed with moderate bilateral testicular degeneration. **a. b.** Seminiferous tubules exhibiting marked atrophy, evident vacuolization, and premature shedding of immature germ cells into the tubular lumen (*). **c**. Epididymal tubules displaying reduced intraluminal sperm content. **d**. Presence of immature germ cells within the epididymal lumen (arrow), indicating disrupted germ cell migration and maturation. Staining: Hematoxylin and eosin (H&E). Scale bar = 100 μm

Histopathological evaluation was performed on testis fixed in 10% buffered formalin, paraffin-embedded, sectioned at 5 μm, and stained with hematoxylin and eosin. Microscopic examination revealed bilateral moderate testicular degeneration, predominantly affecting the seminiferous epithelium. The seminiferous tubules exhibited marked reduction in both tubular diameter and epithelial height, with a predominance of Sertoli cells and a substantial depletion of germ cell populations across most cross-sections (Fig. 2a). Approximately 70% of seminiferous tubules were classified as atrophic (Fig. 2a, b). Tubular vacuolization was prominent, particularly in the basal compartment, and was frequently accompanied by premature exfoliation of immature germ cells into the lumen and extending into the epididymal ducts (Fig. 2c). Notably, despite the widespread degenerative changes, approximately 30% of seminiferous tubules retained evidence of active spermatogenesis, as indicated by the presence of late-stage spermatids and mature spermatozoa within the tubular lumen. Notably, no inflammatory infiltrates were identified in the interstitial tissue, effectively ruling out orchitis and reinforcing a non-inflammatory etiology. The histological features point to a degenerative process of non-infectious origin. While potential contributing factors include hormonal dysregulation, senescence, or prior ischemic insult, the clinical history of unresolved traumatic injury marked by multiple fractures and a lack of recovery despite veterinary care supports trauma as the most plausible underlying cause in this case.

## Discussion and conclusions

The present study documents the first case of moderate bilateral TD in a male jaguarundi, with the diagnosis supported by comprehensive histopathological findings and sperm quality analysis. Baseline descriptions of normal spermatogenesis and testicular architecture in felids have been established through comparative studies in domestic and wild species, including cats, cougars, and jaguars (Costa et al. 2006), providing an essential framework for identifying pathological deviations. TD is a recognized cause of male infertility across mammalian species (McEntee 2012; Koziol and Palmer 2023), including felids (Axnér et al. 1996; Axner 2008). It is typically acquired rather than hereditary (McEntee 2012; Koziol and Palmer 2023), and is characterized by seminiferous tubule atrophy and impaired spermatogenesis, leading to reduced sperm output, testicular shrinkage, and diminished sperm quality (Turner 2007; Koziol and Palmer 2023). In this case, hallmark features such as germ cell depletion, vacuolization, and epithelial disorganization confirmed the diagnosis.

Sperm analysis revealed notably impaired parameters in the affected male, including low progressive motility, poor vigor, and decreased viability. These are indicative of dysfunctional spermatogenesis and align with TD-related deficits described in other mammals (Blanchard et al. 2001; Ortega-Pacheco et al. 2006; Hoflack et al. 2008; Abah et al. 2023).

The presence of teratospermia, a condition commonly seen in felids and characterized by elevated morphological defects (Neubauer et al. 2004; Pukazhenthi et al. 2006; Gutiérrez-Reinoso and García-Herreros 2016; Thongphakdee et al. 2020), further supports this interpretation. Contributing factors such as hormonal imbalance (Müller et al. 2012), diet, stress levels (Swanson et al. 2003), and factors like age (Axnér and Linde Forsberg 2007) are known to influence testicular function and may have exacerbated degeneration. Thus, while TD is rarely reported in wild felids, it may be more prevalent than the literature suggests.

We proposed the diagnosis of moderate bilateral testicular degeneration based on the histopathological classification by Turner (2007). Key histopathological findings in the jaguarundi with TD included the premature shedding of immature germ cells into the seminiferous tubule lumen, loss of normal seminiferous epithelium architecture and the absence of haploid germ cell stages (Koziol and Palmer 2023). Vacuolization of the seminiferous epithelium was also observed, indicating cellular degeneration (Turner 2007; Koziol and Palmer 2023). Some tubules within the testis still contained areas of normal spermatogenesis, highlighting the variable nature of TD, where some parts of the testis remain functional. These findings are consistent with moderate TD, where degenerative changes are prominent but not yet complete, allowing for some sperm production. In addition, the ejaculate still contained a small number of morphologically normal, progressively motile sperm, highlighting the partial functionality of the testis.

The physiological environment within the testis and epididymis, altered by TD, likely impeded normal sperm maturation, rendering the sperm functionally compromised. The plasma membrane integrity and acrosome status of the sperm were also markedly impaired in the affected male, indicating increased susceptibility to environmental challenges. The compromised physiological environment within the reproductive tract likely exacerbated these vulnerabilities, resulting in reduced longevity and decreased fertilizing potential. This finding is consistent with observations in other mammalian species, where sperm from males with testicular degeneration often exhibit increased oxidative stress and reduced membrane stability (Shahat et al. 2020).

The pathogenesis of TD is multifactorial involving several contributing factors, including trauma, fever, malnutrition, hormonal imbalances, and environmental challenges (Foster 2012; Koziol and Palmer 2023). In this case, differential diagnoses considered included orchitis, testicular neoplasia, cryptorchidism, and hormonal imbalances (Axnér et al. 1996; Sigurðardóttir et al. 2001; Prochowska and Niżański 2022; Villalobos-Gomez et al. 2023). However, histopathological analysis revealed characteristic signs of TD, with no evidence of inflammation or neoplastic cells, ruling out orchitis and testicular neoplasia. Additionally, both testicles were present in the scrotal sac, excluding cryptorchidism as a factor. Due to the post-mortem nature of the case and limited clinical history, no endocrine evaluations or diagnostic tests for infectious agents were performed. This limitation restricts the ability to investigate systemic or subclinical factors contributing to the condition; nonetheless, future studies should incorporate evaluations of hormonal and infectious diseases to clarify potential underlying mechanisms.

In mammals, the testis is highly sensitive to disruptions in its microenvironment (Mruk and Cheng 2015; Shahat et al. 2020; Aldahhan and Stanton 2021). Sertoli cells and Leydig cells play crucial roles in supporting spermatogenesis and maintaining testosterone levels (Li et al. 2024). Any disturbance, such as oxidative stress or trauma, can result in a breakdown of this delicate balance, leading to germ cell apoptosis and degeneration of the seminiferous tubules (Shahat et al. 2020; Li et al. 2024). The absence of inflammatory infiltrates was critical for differentiating TD from orchitis.

(Foster 2012; McEntee 2012; Koziol and Palmer 2023), supporting a non-inflammatory etiology, likely due to physical trauma or ischemic damage (McEntee 2012). Environmental factors, such as chronic stress or nutritional deficiencies, may have further compounded the trauma, exacerbating the degenerative process.

The role of oxidative stress in trauma-induced TD cannot be overlooked. Oxidative stress, caused by an overproduction of reactive oxygen species (ROS), can lead to substantial cellular damage, including lipid peroxidation, protein oxidation, and DNA fragmentation (Aitken and Roman 2008; Shahat et al. 2020). In the testis, oxidative stress can impair the blood-testis barrier, disrupt spermatogenesis, and accelerate germ cell apoptosis. Without adequate antioxidant defenses, the buildup of oxidative damage can exacerbate the degeneration of testicular tissue. Furthermore, the stress response, particularly the release of cortisol, can inhibit testosterone production, which is essential for normal spermatogenesis and fertility (Koziol and Palmer 2023).

While studies in domestic felines report a low prevalence of TD (Elcock and Schoning 1984; Foster et al. 1996; Sigurðardóttir et al. 2001; Foster 2012; Chaudhary et al. 2024), the lack of research on wild felids limits our understanding of the true incidence of this condition in natural populations (Thongphakdee et al. 2020). The challenges associated with diagnosing reproductive pathologies in wild felids, such as limited access to samples and the remote habitats of these animals, suggest that TD may be underdiagnosed rather than uncommon. Most available data on felid spermatogenesis are derived from a limited number of comparative studies under controlled conditions (Costa et al. 2006), highlighting the scarcity of pathological data from free-ranging individuals. The present case report highlights the importance of reproductive health studies in wild felids, as conditions like TD can significantly impact population viability, particularly in species with already limited reproductive rates.

A significant limitation of this study is that it reports findings from a single individual, which restricts the generalizability of the observations. However, the rarity of obtaining high-quality reproductive and pathological samples from wild felids, particularly from an elusive and understudied species like the jaguarundi highlights the value of this case.

In conclusion, this study presents the first documented case of moderate bilateral testicular degeneration in a jaguarundi, with significant effects on sperm quality, and functionality. The absence of inflammatory signs and the specific histopathological features observed strongly support a diagnosis of TD likely caused by trauma. This case highlights the need for more focused reproductive studies in wild felids, as TD may be underdiagnosed in these species. Moreover, it underscores the importance of comprehensive reproductive health assessments in conservation programs. Understanding such reproductive pathologies is critical for conservation strategies aimed at preserving the reproductive health and population viability of wild felids.

## Supplementary Information

Below is the link to the electronic supplementary material.

Supplementary Material 1

## Data Availability

No datasets were generated or analysed during the current study.
