# Supplementary material for: Testicular degeneration in a jaguarundi (Puma yagouaroundi): a histological and functional case study
Source: Vet Res Commun. 2026 Feb 9;50(2):151. doi: 10.1007/s11259-026-11098-3 (PMC12886296; doi:10.1007/s11259-026-11098-3)
Supplement: Supplementary file 1 — Supplementary Material 1 [file 11259_2026_11098_MOESM1_ESM.docx]

**Supplementary material**

**Suppl. Table 1** Morphology of epididymal sperm from jaguarundi male with testis degeneration.

| **Sperm characteristics** | **Mean ± standard error** |
| --- | --- |
| **Normal sperm** | 31.5 ± 2.12 |
| **Head defects total** | 12 ± 5.23 |
| Microcephaly | 2.5 ± 2.12 |
| Macrocephaly | 1.5 ± 0.71 |
| Spindle head | 4.0 ± 2.83 |
| Bulb head | 2.5 ± 2.12 |
| Double head | 1.5 ± 2.12 |
| **Detached head** | 3.5 ± 0.71 |
| **Acrosomal defects** | 3.2 ± 1.54 |
| **Middle piece defect** | 12.3 ± 2.12 |
| **Midpiece broken at neck** | 2.0 ± 0.0 |
| **Tail defects total** | 13 ± 4.24 |
| Distal coiled tail | 6.0 ± 1.41 |
| Proximal coiled tail | 7.0 ± 2.83 |
| **Proximal cytoplasmic droplet** | 15.0 ± 1.41 |
| **Distal cytoplasmic drop** | 7.5 ± 3.54 |


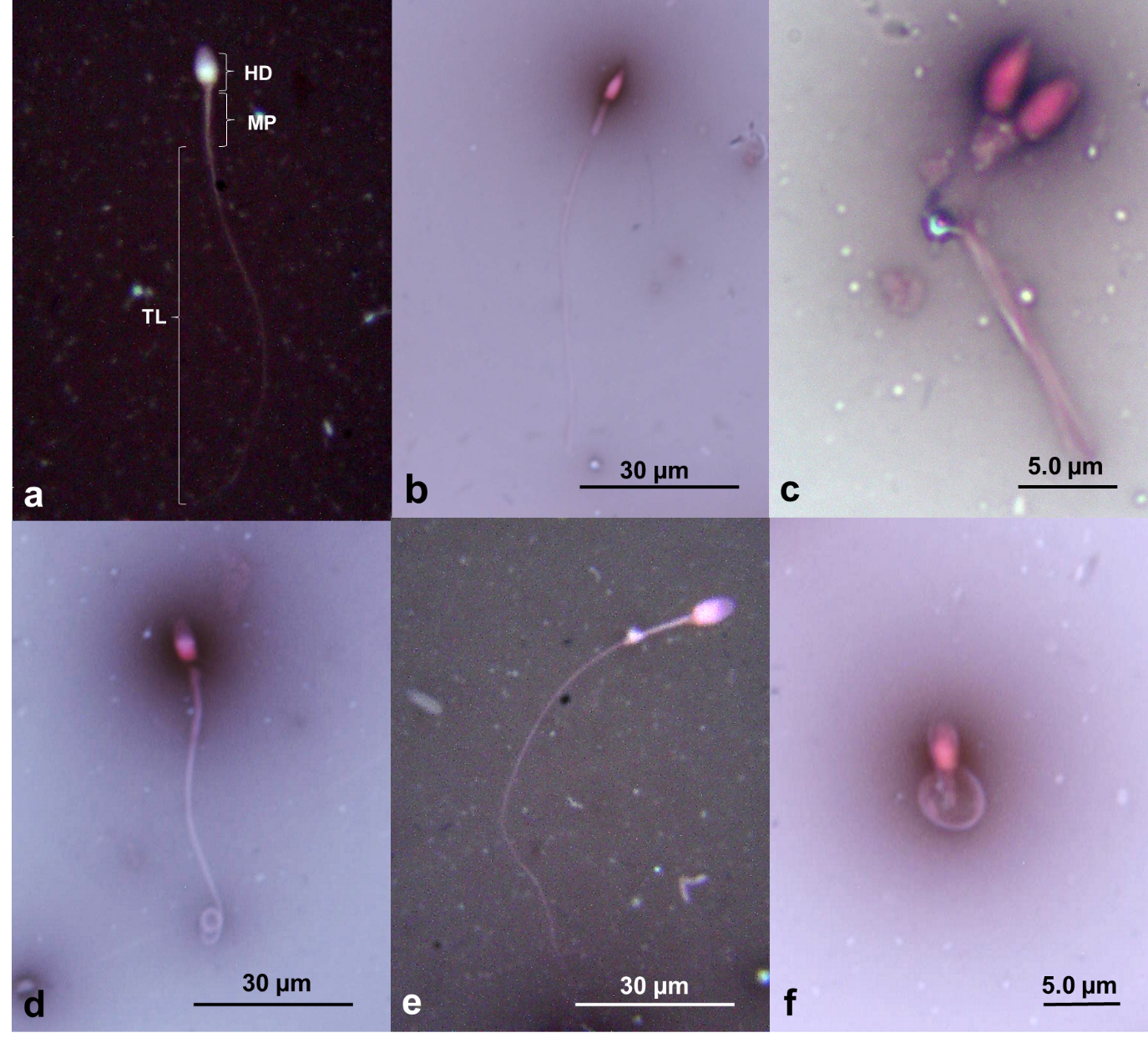


**Suppl. Fig. 1.** Micrographs of fresh sperm cells from jaguarundi (*P. yagouaroundi*). a. normal sperm - HD: head; MP: midpiece; TL: tail (F). b: Spindle head (F). c. Broke double head. d. Coiled tail. e. Proximal cytoplasmic drop. f. Tightly coiled tail. Eosin-nigrosin stain.

**Suppl. Fig. 2.** Functional characteristics of epididymal sperm from jaguarundi male during incubation at 37 °C (thermo-resistance test).
